# Transient Elastography for Significant Liver Fibrosis and Cirrhosis in Chronic Hepatitis B: A Meta-Analysis

**DOI:** 10.1155/2018/3406789

**Published:** 2018-05-24

**Authors:** Xiaolong Qi, Min An, Tongwei Wu, Deke Jiang, Mengyun Peng, Weidong Wang, Jing Wang, Chunqing Zhang, on behalf of the CHESS Study Group

**Affiliations:** ^1^Department of Gastroenterology, Shandong Provincial Hospital Affiliated to Shandong University, Jinan, China; ^2^CHESS, Hepatic Hemodynamic Lab, Institute of Hepatology, Nanfang Hospital, Southern Medical University, Guangdong Provincial Research Center for Liver Fibrosis, Guangzhou, China; ^3^The Second School of Clinical Medicine, Southern Medical University, Guangzhou, China; ^4^Department of Hepatobiliary Disease, The Affiliated (T.C.M) Hospital of Southwest Medical University, Luzhou, China; ^5^Department of Hepatobiliary Surgery, Shunde Hospital, Southern Medical University, Foshan, China

## Abstract

**Background:**

The hepatitis B virus infection is a global health issue and the stage of liver fibrosis affects the prognosis in patients with chronic hepatitis B (CHB). We performed the meta-analysis describing diagnostic accuracy of transient elastography (TE) for predicting CHB-related fibrosis.

**Methods:**

We performed an adequate literature search to identify studies that assessed the diagnostic accuracy of TE in CHB patients using biopsy as reference standard. Hierarchical summary receiver-operating curves model and the bivariate mixed-effects binary regression model were applied to generate summary receiver-operating characteristic curves and pooled estimates of sensitivity and specificity.

**Results:**

The area under the summary receiver-operating curve for significant fibrosis and cirrhosis was 0.86 (95% confidence interval (CI): 0.83–0.89) and 0.92 (95% CI: 0.90–0.94), respectively. The sensitivity, specificity, and diagnostic odds ratio of TE for significant fibrosis were 0.78 (95% CI: 0.73–0.81, *p* < 0.01; *I*^2^ = 85.59%), 0.81 (95% CI: 0.77–0.84, *p* < 0.01; *I*^2^ = 88.20%), and 14.44 (95% CI: 10.80–19.31, *p* < 0.01; *I*^2^ = 100%) and for cirrhosis were 0.84 (95% CI: 0.80–0.88, *p* < 0.01; *I*^2^ = 76.67%), 0.87 (95% CI: 0.84–0.90, *p* < 0.01; *I*^2^ = 90.89%), and 36.63 (95% CI: 25.38–52.87, *p* < 0.01; *I*^2^ = 100%), respectively. The optimal cut-off values of TE were 7.25 kPa for diagnosing significant fibrosis and 12.4 kPa for diagnosing cirrhosis, respectively.

**Conclusion:**

TE is of great value in the detection of patients with CHB-related cirrhosis but has a suboptimal accuracy in the detection of significant fibrosis.

## 1. Introduction

Chronic hepatitis B virus infection continues to be a major public health issue worldwide with the prevalence of 3.61% [1]. As well known, liver fibrosis, one of the main prognostic factors in chronic hepatitis B (CHB), was associated with the risk of developing cirrhosis and cirrhosis-related complications [2, 3]. Therefore, liver fibrosis stage plays one of the most important roles in diagnostic and prognostic assessments in patients with CHB.

Liver biopsy (LB), as invasive in nature with related risks, is the gold standard for fibrosis assessment. However, LB is associated with obvious patient discomfort and risk of complications ranging from pain to more serious events with hospitalization rate of 1.4–3.2% [4] and mortality varying from 0.0088 to 0.3% [5]. Besides, LB provides only a quite small part of the organ, and thus there is a risk that the small part might not be representative for the live fibrosis in the whole liver [6].

Noninvasive methods of assessing fibrosis and cirrhosis were urgently needed, and serologic tests and novel imaging techniques were recently developed [7, 8]. Most of these studied focused on whether noninvasive methods can accurately detect minimal (F0-1), significant (≥F2), or advanced (≥F3-4) fibrosis based on the METAVIR score [9]. Transient elastography (TE), also known as FibroScan, was a device and a well-validated method with advantages of a short procedure time (<5 min), immediate results, and the ability to perform the test at the bedside or in an outpatient clinic [10]. Compared with blood tests, TE has a similar performance to predict significant fibrosis (SF) and higher accuracy to identify cirrhosis [11]. Measurement of liver fibrosis without biopsy is very tempting. In spite of the fact that recommendations suggested that noninvasive tests were still not ready to replace LB [12, 13], TE has become widely present in clinical practice. The accuracy of TE for detection of fibrosis has been assessed extensively in a variety of liver diseases [14–17]. However, it was reported that the presence of an IQR/M > 30% and liver stiffness median ≥7.1 kPa lead to a lower accuracy determined by the area under receiver-operating curve (AUROC) and these cases were considered “poorly reliable” [18]. Another study also indicated that there was a significant discrepancy in up to 20% of cases cirrhosis between different TE devices [19].

In the study, we performed an independent meta-analysis of the diagnostic accuracy of TE for predicting significant liver fibrosis (F2–4 versus F0-1) and cirrhosis (F4 versus F0–3) in CHB patients.

## 2. Methods

### 2.1. Literature Search Strategy

PubMed, Web of Science, and EMBASE database were searched to October 10, 2016, as well as Wanfang database and China National Knowledge Infrastructure. The search strategy was “FibroScan or transient elastography” in combination with “liver fibrosis assessment,” “significant fibrosis or cirrhosis or advanced liver fibrosis,” and “liver stiffness measurement.” All eligible studies were retrieved and their reference lists were checked for additional relevant publications.

### 2.2. Inclusion Criteria

All diagnostic cross-sectional studies, cohort studies, and randomized studies that compared TE accuracy with biopsy in diagnosis fibrosis grade were eligible for inclusion. Studies that met all the following criteria were included: (i) studies which reported that all patients had undergone biopsy and TE; (ii) having enough data to create 2 × 2 table of test performance (with numbers of true and false positives and negatives); and (iii) studies which reported the method of definition of the fibrosis grade.

### 2.3. Exclusion Criteria

The exclusion criteria were as follows: (i) the patients belonging to the pediatric population, hepatitis C/hepatitis B virus coinfected patients, mixed chronic liver disease patients (but not CHB and nonalcoholic fatty liver disease), and liver/kidney transplant patients; (ii) studies that were clearly extensions of previously published cohorts; and (iii) studies unable to obtain sufficient data for statistical analysis.

### 2.4. Methodological Assessment

Methodological quality was assessed by the Quality Assessment of Diagnostic Accuracy Studies-2 (QUADAS-2) tool. QUADAS-2 was designed to assess the internal and external validity. Any differences between two authors were resolved with discussion between the two review authors and the third author was final arbiter.

### 2.5. Data Extraction and Management

As for each study, the following information was extracted: year of publication, study design, sample size, presence of HIV coinfection, the QUADAS-2 methodological items, prevalence of each fibrosis stage on biopsy, along with total prevalence of SF and cirrhosis, interval between biopsy and TE, size of biopsy sample, type of scoring system used for histology (METAVIR versus other), and AUROC. Two authors performed the data extraction independently. Disagreement was resolved with discussion between the two review authors, with a third author as final arbiter.

### 2.6. Statistical Analysis and Data Synthesis

Initial analysis was performed with the Review Manager (RevMan) 5.0. Stata 12.0 was used for meta-analysis of diagnostic accuracy studies, to compute the pooled sensitivity and specificity and to plot the summary receiver-operating characteristics curve (SROC) with summary point and corresponding 95% confidence interval (CI). Regression analysis was performed by Stata 12.0, with each time point providing another covariate to verify the influence of the chosen covariate on the accuracy estimates. We used hierarchical SROC model and the bivariate random efforts model to produce SROC and pooled estimates of sensitivity and specificity. We performed Fagan test to detect clinical significant by Stata 12.0. Heterogeneity was assessed with the inconsistency index (*I*^2^) and *I*^2^ values over 50% indicated substantial heterogeneity. Heterogeneity from threshold effect was explored by meta-disc 1.4.

## 3. Results

### 3.1. Search Results

**Figure 1:**
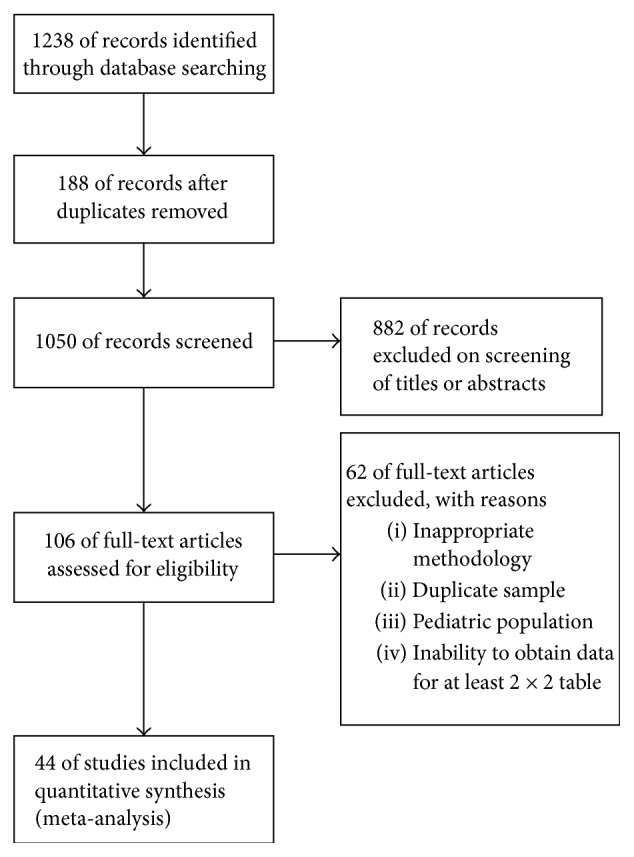
Flow diagram of study selection process.

1238 articles were obtained and 188 were excluded for duplicates. 882 were excluded based on title and abstracts, and full-text copies of 106 studies were obtained and assessed for eligibility. Furthermore, 62 were excluded for inappropriate methodology, duplicate sample, pediatric population, or inability to obtain data for at least 2 × 2 table. Finally, a total of 44 articles comprising 45 studies were enrolled in the meta-analysis (Figure 1).

### 3.2. Characteristics of Included Studies

The overall prevalence of SF (F2–4) and cirrhosis (F4) ranged from 14.8% to 92.3% and from 1.1% to 69.2%, respectively. Reported AUROCs for SF diagnosis ranged from 0.614 to 0.98 (Table 1).

As shown in Table 1, only Miailhes et al. (*N* = 59) reported HIV coinfected patients [20]. In sixteen studies (*N* = 2664), LB was assessed with a histological score other than METAVIR [21–36]. In eight studies (*N* = 1109), mean length of biopsy sample was ≥20 mm [22, 34, 37–42]. Besides, in nineteen studies (*N* = 1358), data on time interval between biopsy and TE were not obtained [11, 21, 23, 25, 27, 28, 32–34, 39, 40, 42–47]. Three studies did not report cirrhosis (F4) [24, 35, 48]. Only four studies were retrospective [31, 48–50].

**Figure 2:**
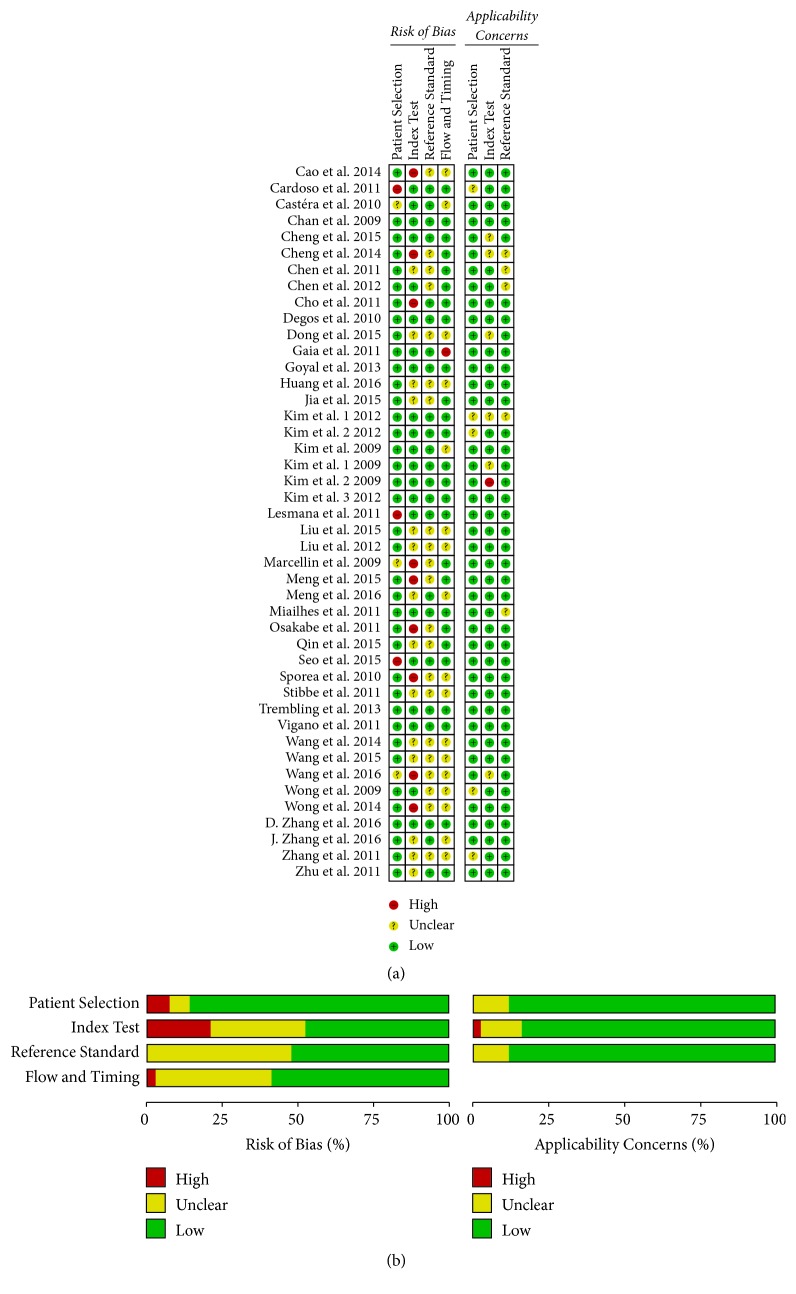
Summary of methodological quality of 44 studies according to Quality Assessment of Diagnostic Studies-2 (QUDAS-2) tool. (a) Overall and (b) study-level of bias.

As presented in Figure 2, the results of methodological quality assessment based on the QUADAS-2 scale were depicted for all of the 44 eligible studies. The majority of the methodological concern lies within the index test, because TE in ten studies interpreted with knowledge of the results of the biopsy [24, 29, 33, 39, 46, 48, 51–54] and TE in one study was conducted with assistance by a time-motion ultrasound image [40]. Another possible issue was addressed in patient selection that participants might be enrolled consecutively with confirmed diagnosis in three studies [31, 50, 55]. Both of these concerns might be located in heterogeneity and sensitivity analyses.

### 3.3. Diagnosis of SF

**Figure 3:**
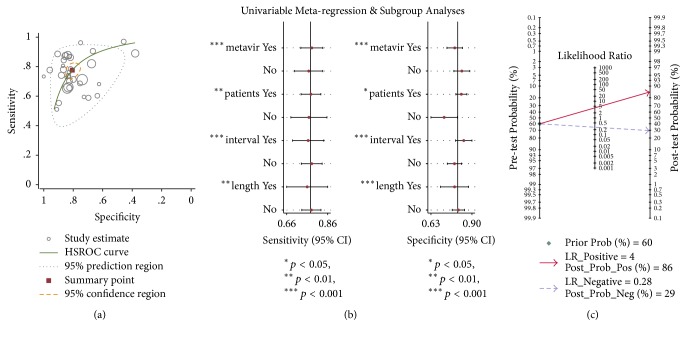
Meta-analysis of 32 studies that assessed the diagnosis accuracy of significant fibrosis based on transient elastography. (a) A summary receiver-operating characteristic (SROC) plot of transient elastography for detection of significant liver fibrosis (METAVIR F2–F4). (b) Regression analysis of studies whether reported with METAVIR score on the next day of biopsy or with sample size ≥ 20 cm for significant liver fibrosis. (c) Detection of clinical significance for significant liver fibrosis (METAVIR F2–F4) based on Fagan test. Heterogeneity was generated if *p* < 0.01 in sensitivity or specificity separately. However, joint *p* value was generated synthesisly for analysis of both sensitivity and specificity.

We included 35 studies (*N* = 6,202) in the analysis for SF (F2–F4) [15–23, 25–27, 29–35, 37–40, 43, 56–59]. Summary representation of the overall analysis was presented in Figure 3(a) and Supplementary Figure 1. Sensitivity and specificity ranged from 51 to 97% and 38 to 100%, respectively (Supplementary Figure 1).

The area under SROC for SF was 0.86 (95% CI: 0.83–0.89) (Figure 3(a)). The meta-analysis summary estimate indicated pooled sensitivity of 0.78 (95% CI: 0.73–0.81, *p* < 0.01; *I*^2^ = 85.59%), specificity of 0.81 (95% CI: 0.77–0.84, *p* < 0.01; *I*^2^ = 88.20%) (Supplementary Figure 1(A)), positive likelihood ratio (LR+) of 4.01 (95% CI: 3.31–4.84, *p* < 0.01; *I*^2^ = 86.27%), negative likelihood ratio (LR−) of 0.28 (95% CI: 0.23–0.33, *p* < 0.01; *I*^2^ = 81.95%) (Supplementary Figure 1(B)), diagnostic score (DS) of 2.67 (95% CI: 2.38–2.96, *p* < 0.01; *I*^2^ = 71.57%), and diagnostic odds ratio (DOR) of 14.44 (95% CI: 10.80–19.30, *p* < 0.01; *I*^2^ = 100%) (Supplementary Figure 1(C)). However, it must be carefully considered as they were not pooled from studies with identical TE threshold. Overall, there was heterogeneity as graphically illustrated on the forest plot in Supplementary Figure 1. The cut-off value for SF (F2–4) ranged from 5.2 to 10.3 kPa with a mean value of 8.6 kPa and a median of 7.25 kPa.

As shown in Figure 3(b) and Table 2, in the analysis of LB-related factors with an impact on accuracy, there was no significant difference (joint *p* = 0.47 for classification criteria; joint *p* = 0.29 for interval time; joint *p* = 0.77 for average sample size). 26 studies conducted in Asian presented a better both pooled sensitivity (0.78, 95% CI: 0.73–0.82) and specificity (0.83, 95% CI: 0.79–0.87) than in Caucasian (joint *p* = 0.03).

As presented in Figure 3(c), it was indicated that posttest probability of LR+ increased to 86% and LR− decreased to 29% after TE was performed based on Fagan test.

### 3.4. Diagnosis of Cirrhosis

41 studies were included in the cirrhotic analysis with a total of 7,205 patients, as four studies did not have any cases of liver cirrhosis (METAVIR F4) [21, 24, 35, 48]. The overall prevalence of METAVIR F4 and the AUROCs in the included studies ranged from 5% to 69.2% and from 0.80 to 0.98 (Table 1), respectively.

**Figure 4:**
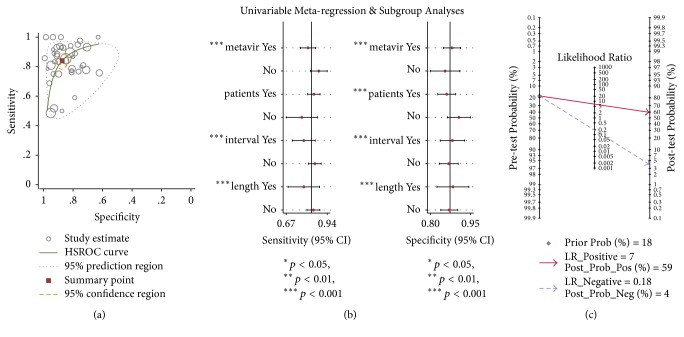
Meta-analysis of 37 studies that assessed the diagnosis accuracy of cirrhosis based on transient elastography. (a) A summary receiver-operating characteristic (SROC) plot of transient elastography for detection of cirrhosis (METAVIR F4). (b) Regression analysis of studies whether reported with METAVIR score on the next day of biopsy or with sample size ≥ 20 cm for cirrhosis. (c) Detection of clinical significance for cirrhosis (METAVIR F4) based on Fagan test.

Summary representation of the overall analysis was shown in Figure 4(a). The area under the SROC for liver cirrhosis was 0.92 (95% CI: 0.90–0.94). Sensitivity ranged from 49% to 100%, much more widely than specificity which ranged from 62% to 99% (Supplementary Figure 2). The meta-analysis summary estimate covered the pooled sensitivity of 0.84 (95% CI: 0.80–0.88, *p* < 0.01; *I*^2^ = 76.67%), specificity of 0.87 (95% CI: 0.84–0.90, *p* < 0.01; *I*^2^ = 90.89%) (Supplementary Figure 2(A)), LR+ of 6.66 (95% CI: 5.34–8.31, *p* < 0.01; *I*^2^ = 84.77%), LR− of 0.18 (95% CI: 0.14–0.23, *p* < 0.01; *I*^2^ = 80.80%) (Supplementary Figure 2(B)), DS of 3.60 (95% CI: 3.23–3.97, *p* < 0.01; *I*^2^ = 66.54%), and DOR of 36.63 (95% CI: 25.38–52.87, *p* < 0.01; *I*^2^ = 100%), respectively (Supplementary Figure 2(C)). Again, these measures must be carefully considered without identical TE thresholds. The cut-off value for cirrhosis ranged from 9 kPa to 18.2 kPa with both a mean value and a median of 12.4 kPa.

As shown in Figure 4(b) and Table 3, although summary sensitivity was lower and summary specificity was higher in studies with METAVIR score (sensitivity: 0.82, 95% CI: 0.77–0.87; specificity: 0.88, 95% CI: 0.85–0.91), TE performed on the next day of LB (sensitivity: 0.79, 95% CI: 0.71–0.86; specificity: 0.88, 95% CI: 0.84–0.93), and average sample length ⩾ 20 mm (sensitivity: 0.79, 95% CI: 0.69–0.89; specificity: 0.88, 95% CI: 0.83–0.94), respectively, no statistical significance was detected (joint *p* = 0.17 for classification criteria; joint *p* = 0.21 for interval time; joint *p* = 0.47 for average sample size). Besides, pooled sensitivity and specificity were without significant difference (joint *p* = 0.12) between Caucasian (sensitivity: 0.78, 95% CI: 0.67–0.88; specificity: 0.91, 95% CI: 0.86–0.95) and Asian (sensitivity: 0.86, 95% CI: 0.81–0.90; specificity: 0.86, 95% CI: 0.83–0.89).

In addition, based on Fagan test, it was illustrated that posttest probability of LR+ and LR− rose and declined to 59% and 4%, respectively (Figure 4(c)).

### 3.5. Publication Bias

The results of publication bias analysis were performed with Stata in Supplementary Figure 3. No significant publication bias was detected according to Deeks figures for SF (*p* = 0.26). However, there was bias among 41 studies enrolled in analysis of TE for cirrhosis (*p* = 0.02), which might result from the positive results of all 41 studies.

## 4. Discussion

TE can provide a reliable detection of liver fibrosis in patients with CHB and thus has been recommended by the American Association for the Study of Liver Diseases (AASLD) and European Association for the Study of the Liver (EASL) [60, 61]. This meta-analysis was conducted in a total of 7,808 CHB patients to summarize the diagnostic accuracy of TE for CHB-related SF, with optimal statistical method SROC. In addition, regression analysis was carried out to further explore sources of heterogeneity.

In our study, TE performed well in both SF (F2–4) and cirrhosis (F4) with pooled sensitivity of 78% and 84%, summary specificity of 81% and 87%, DOR of 14.44 and 36.63, LR+ of 4.01 and 6.66, LR− of 0.28 and 0.18, respectively. Study by Li et al. [62] with hierarchical SROC model was also performed in CHB patients, with summary sensitivity and specificity for SF (F2–4) and cirrhosis (F4) of 80% and 86%, 82%, and 88%, however, without DOR, LR+ and LR−. Interestingly, the pooled specificity for diagnosis SF (F2–4) and cirrhosis (F4) in both studies were higher than summary sensitivity, which suggested that the currently cut-off values of TE performed better in excluding diseases rather than confirming diseases. Furthermore, the areas under the SROC were 0.86 for SF (F2–4) and 0.92 for cirrhosis (F4), respectively, which indicated that TE was performed well in staging fibrosis in CHB patients. In addition, TE performed better for cirrhosis than SF with a higher value of AUC, sensitivity, specificity, DOR, LR+, and a lower value of LR−. Although the diagnostic accuracy was higher for cirrhosis, TE could also increase the diagnostic accuracy for SF based on Fagan test with increased LR+ and decreased LR−.

The higher TE values were used to confirm diagnosis, while the lower one was used to exclude the false positive diagnosis. However, if the TE value located between the values for rule in and rule out, biopsy was then recommended. Based on the descriptive statistics of enrolled studies, the cut-off values for diagnosing SF (F2–4) and cirrhosis (F4) ranged from 5.2 to 10.3 kPa and 9 to 18.2 kPa, respectively. The optimal cut-off values of TE in CHB patients in our study were 7.25 kPa for SF (F2–4) and 12.4 kPa for cirrhosis (F4). In the previous meta-analysis by Li et al., the weighted mean cut-off values of TE were comparable with 7.2 kPa for SF (F2-4) and 12.2 kPa for cirrhosis (F4) [62]. However, since there was no optimal statistical method to pool different cut-off values in individual studies, the optimal cut-off values in our meta-analysis were simply summarized as median, which could eliminate the impact resulting from the maximum and minimum values that was better than the mean value in previous study [62].

Elevated ALT levels might affect the predictive accuracy of TE [16, 24, 45, 50, 55, 56]; however, the study by Cardoso et al. reported that the use of TE cut-off values adjusted to ALT level did not improve the performance of liver stiffness in CHB patients [49]. Although elevated ALT might be the most important confounder on liver stiffness measurement, the synthesis analysis of ALT elevation could not be conducted due to insufficient data. Therefore, it would be beneficial if more clinical studies focused on the correlation between ALT elevation and TE in CHB patients.

One of the main limitations in this meta-analysis was the significant heterogeneity of the included studies. Spearman correlation coefficient for SF and cirrhosis were 0.055 (*p* = 0.755) and 0.057 (*p* = 0.723), and no threshold effect was presented. Therefore, regression analysis was carried out. Besides, TE value could be applied as diagnosis criteria for both SF and cirrhosis in Asian. However, for Caucasian, it was noted that TE was valid to diagnosis of cirrhosis, while it was less precise for SF. Unfortunately, the regression analysis was not conducted owing to the small size of HIV- and non-HIV-coinfected patients. It should be noted that the overlapped cut-off values from included studies might also result in the heterogeneity.

In conclusion, TE is of great value for detection CHB-related cirrhosis, however, with a suboptimal performance in detection of SF. Further studies should focus on the TE cut-off value and the effect of ALT elevation in patients with CHB.

## Figures and Tables

**Table 1:** Characteristics of the included studies.

Author	Study type	Year	HIV/HBV	METAVIR	Biopsy size	Biopsy to TE time (days)	Sample	Prevalence F2–F4	Prevalence F4	TE cut-off	AUROC
Cao et al.	Prospective	2014	NO	YES	>=15 mm and >=6 portal tracts	NA	162	0.61	0.12	7.3/17.5	NA/NA
Cardoso et al.	Retrospective	2011	NO	YES	>=15 mm and/or >=6 portal tracts	1	202	0.421	0.079	7.2/11	0.867/0.935
Castéra et al.	Prospective	2010	NO	YES	>=16 mm	NA	60	0.73	0.25	7.1/9.6	0.76/0.89
Chan et al.	Prospective	2009	NO	YES	>=15 mm and >=6 portal tracts	28	136	NA	0.25	NA/9	NA/0.93
Chen et al.	Retrospective	2011	NO	YES	>=15 mm	7	213	0.479	0.15	7.0/13.0	0.916/0.971
Chen et al.	Prospective	2012	NO	YES	>=15 mm and >=10 portal tracts	7	315	0.771	0.235	NA/10.4	NA/0.88
Cheng et al.	Prospective	2015	NO	YES	>=10 mm and >=8 portal tracts	1	459	0.61	0.152	7.2/18.2	0.82/0.87
Cheng et al.	Prospective	2014	NO	NO	>=15 mm and >=6 portal tracts	1	99	0.54	NA	8.15/NA	0.896/NA
Cho et al.	Prospective	2011	NO	YES	>=15 mm	1	121	0.727	0.074	7.8/14.0	0.849/0.867
Degos et al.	Prospective	2010	NO	YES	>=18 mm	1	284	0.415	0.102	5.2/12.9	0.78/0.90
Dong et al.	Prospective	2015	NO	NO	>=15 mm and >=6 portal tracts	NA	81	0.604	0.098	10.3/9.4	0.753/0.873
Gaia et al.	Prospective	2011	NO	YES	>=20 mm	120	70	0.53	0.31	7.2/10.6	0.674/0.763
Goyal et al.	Prospective	2013	NO	YES	>=15 mm and >=6 portal tracts	38	357	0.792	0.059	6.0/11.0	0.84/0.93
Huang et al.	Prospective	2016	NO	NO	>=15 mm	NA	263	0.148	0.011	8/NA	0.911/NA
Jia et al.	Prospective	2015	NO	YES	>=10 mm and >=8 portal tracts	NA	469	0.612	0.122	7.3/10.7	0.82/0.90
Kim et al. 1	Prospective	2012	NO	NO	>=20 mm	1	194	0.845	0.387	8.8/14.1	0.873/0.910
Kim et al. 2	Prospective	2012	NO	YES	>=20 mm	1	170	0.712	0.276	8.0/14.0	0.937/0.963
Kim et al.	Prospective	2009	NO	YES	>=10 mm and >=10 portal tracts	1	91	NA	0.692	NA/10.3	NA/0.803
Kim et al. 1	Prospective	2009	NO	YES	>=6 portal tracts	1	130	0.923	0.515	NA/10.1	NA/0.840
Kim et al. 2	Prospective	2009	NO	YES	>=15 mm	NA	91	0.868	0.396	NA/NA	0.837/0.913
Kim et al. 3	Prospective	2012	NO	NO	>=15 mm	1	150	0.847	0.453	6.0/9.4	NA/NA
Lesmana et al.	Retrospective	2011	NO	YES	>=15 mm and >=5 portal tracts	1	117	0.624	NA	5.85/NA	0.614/NA
liu et al.	Prospective	2015	NO	NO	>=8 portal tracts	NA	115	0.53	0.15	8.50/11.75	0.838/0.914
Liu et al.	Prospective	2012	NO	NO	>=10 mm	NA	134	0.43	0.11	7.60/13.20	0.93/0.96
Marcellin et al.	Prospective	2009	NO	YES	>=6 portal tracts	1	173	0.503	0.081	7.2/11.0	0.81/0.93
Meng et al.	Prospective	2015	NO	YES	>=12 mm and >=6 portal tracts	2	287	0.488	0.157	8.85/17.05	0.909/0.815
Meng et al.	Prospective	2016	NO	NO	>=15 mm	7	168	NA	0.15	15.1	0.927
Miailhes et al.	Prospective	2011	YES	YES	>=10 mm	3	59	0.61	0.203	5.9/9.4	0.85/0.96
Osakabe et al.	Prospective	2011	NO	YES	>=15 mm and >=8 portal tracts	30	51	0.882	0.275	7.1/16.0	0.844/0.93
Qin et al.	Prospective	2015	NO	NO	NA	1	152	0.68	0.07	8.2/13.1	0.752/0.973
Seo et al.	Retrospective	2015	NO	NO	>=15 mm	90	567	0.72	0.2	7.8/11.6	0.774/0.902
Sporea et al.	Prospective	2010	NO	YES	>=20 mm and >=8 portal tracts	NA	140	0.764	0.05	7/13.6	0.658/0.974
Stibbe et al.	Prospective	2011	NO	YES	>=20 mm	NA	48	0.458	0.104	7.0/14.0	NA/0.89
Trembling et al.	Prospective	2013	NO	YES	>=20 mm	1	182	0.626	0.198	NA/11.85	NA/O.95
Vigano et al.	Prospective	2011	NO	YES	>=20 mm	NA	125	0.53	0.16	6.2/13.1	NA/NA
Wang et al.	Prospective	2015	NO	NO	>=15 mm and >=6 portal tracts	NA	142	0.585	0.092	8.15/13.95	0.897/0.968
Wang et al.	Prospective	2014	NO	NO	>=15 mm	NA	80	0.7	0.1125	7.3/12.4	0.865/0.944
Wang et al.	Prospective	2016	NO	NO	NA	NA	127	0.76	0.24	NA/15.2	NA/0.805
Wong et al.	Prospective	2009	NO	YES	>=15 mm and >=6 portal tracts	NA	134	0.78	0.24	NA/13.4	NA/0.89
Wong et al. Tr-c	Prospective	2014	NO	YES	>=15 mm and >=6 portal tracts	NA	238	0.693	0.235	NA/10	NA/0.9
Wong et al. Va-c	Prospective	2014	NO	YES	>=15 mm and >=6 portal tracts	NA	85	0.565	0.259	NA/10	NA/0.87
Zhang et al.	Prospective	2016	NO	NO	>=22 mm	7	180	0.72	0.18	7.5/10.6	0.813/0.799
Zhang et al.	Prospective	2016	NO	NO	>=15 mm	NA	124	0.54	NA	6.95	0.732
Zhang et al.	Prospective	2011	NO	NO	>=15 mm and >=6 portal tracts	NA	88	0.671	0.159	7.25/12.40	0.857/0/948
Zhu et al.	Prospective	2011	NO	YES	>=15 mm and >=6 portal tracts	1	175	NA	0.166	7.9/13.8	NA/0.98

AUROC, area under the receiver-operating curve; TE, transient elastography; HIV/HBV, hepatitis B and HIV-coinfected patients; METAVIR, liver biopsy assessed according to METAVIR or not; TE cut-off, TE cut-off used to predict; NA, data not available.

**Table 2:** Results of meta-regression for significant fibrosis.

Covariate	Number	Pooled sensitivity	*p* value	Pooled specificity	*p* value	Joint *p* value
Classification criteria						
METAVIR score	21	0.78 (0.75–0.83)	<0.01	0.79 (0.73–0.84)	<0.01	0.47
Non-METAVIR score	14	0.77 (0.70–0.83)		0.83 (0.78–0.89)		
Interval time						
On the next day of liver biopsy	11	0.76 (0.69–0.84)	<0.01	0.85 (0.79–0.90)	<0.01	0.29
More than one day after liver biopsy	24	0.78 (0.73–0.83)		0.78 (0.74–0.83)		
Average sample size						
*⩾*20 mm	7	0.76 (0.66–0.86)	<0.01	0.79 (0.69–0.88)	<0.01	0.77
Not *⩾* 20 mm	28	0.78 (0.74–0.82)		0.81 (0.77–0.85)		
Region						
Asian	26	0.78 (0.73–0.82)	<0.01	0.83 (0.79–0.87)	0.04	0.03
Caucasian	9	0.77 (0.68–0.85)		0.72 (0.63–0.80)		

**Table 3:** Results of meta-regression for cirrhosis.

Covariate	Number	Pooled sensitivity	*p* value	Pooled specificity	*p* value	Joint *p* value
Classification criteria						
METAVIR score	28	0.82 (0.77–0.87)	<0.01	0.88 (0.85–0.91)	<0.01	0.17
Non-METAVIR score	13	0.89 (0.83–0.94)		0.86 (0.80–0.91)		
Interval time						
On the next day of liver biopsy	13	0.79 (0.71–0.86)	<0.01	0.88 (0.84–0.93)	<0.01	0.21
More than one day after liver biopsy	28	0.86 (0.82–0.90)		0.87 (0.83–0.90)		
Average sample size						
*⩾*20 mm	8	0.79 (0.69–0.89)	<0.01	0.88 (0.83–0.94)	<0.01	0.47
Not *⩾* 20 mm	33	0.85 (0.81–0.89)		0.87 (0.84–0.90)		
Region						
Asian	31	0.86 (0.81–0.90)	<0.01	0.86 (0.83–0.89)	<0.01	0.12
Caucasian	10	0.78 (0.67–0.88)		0.91 (0.86–0.95)		

## Abbreviations

CHB: Chronic hepatitis B
CI: Confidence interval
DOR: Diagnostic odds ratio
LB: Liver biopsy
LR: Likelihood ratio
QUADAS-2: Quality Assessment of Diagnostic Accuracy Studies-2
SF: Significant fibrosis
SROC: Summary receiver-operating curves
TE: Transient elastography
AUROC: Area under receiver-operating curve.

## Supplementary Materials

Supplementary Materials Supplementary Figure 1: meta-analysis of 32 studies that assessed the diagnosis accuracy of significant fibrosis (METAVIR F2–F4) based on transient elastography. A Forest plot of (A) sensitivity and specificity, (B) positive and negative likelihood ratio, and (C) diagnostic score (DS) and diagnostic odds ratio (DOR) for significant liver fibrosis (METAVIR F2–F4). Supplementary Figure 2: meta-analysis of 37 studies that assessed the diagnosis accuracy of cirrhosis (METAVIR F4) based on transient elastography. A Forest plot of (A) sensitivity and specificity, (B) positive and negative likelihood ratio, and (C) DS and DOR for cirrhosis (METAVIR F4). Supplementary Figure 3: Deeks' Funnel Plot Asymmetry Test for (A) significant fibrosis (METAVIR F2–F4) and (B) cirrhosis (METAVIR F4).
