# Supplementary material for: Transient Elastography for Significant Liver Fibrosis and Cirrhosis in Chronic Hepatitis B: A Meta-Analysis
Source: Can J Gastroenterol Hepatol. 2018 May 24;2018:3406789. doi: 10.1155/2018/3406789 (PMC5994263; doi:10.1155/2018/3406789)
Supplement: Supplementary Materials — Supplementary Figure 1: meta-analysis of 32 studies that assessed the diagnosis accuracy of significant fibrosis (METAVIR F2–F4) based on transient elastography. A Forest plot of (A) sensitivity and specificity, (B) positive and negative likelihood ratio, and (C) diagnostic score (DS) and diagnostic odds ratio (DOR) for significant liver fibrosis (METAVIR F2–F4). Supplementary Figure 2: meta-analysis of 37 studies that assessed the diagnosis accuracy of cirrhosis (METAVIR F4) based on transient elastography. A Forest plot of (A) sensitivity and specificity, (B) positive and negative likelihood ratio, and (C) DS and DOR for cirrhosis (METAVIR F4). Supplementary Figure 3: Deeks' Funnel Plot Asymmetry Test for (A) significant fibrosis (METAVIR F2–F4) and (B) cirrhosis (METAVIR F4). [file 3406789.f1.zip › 3406789.f6.pdf]

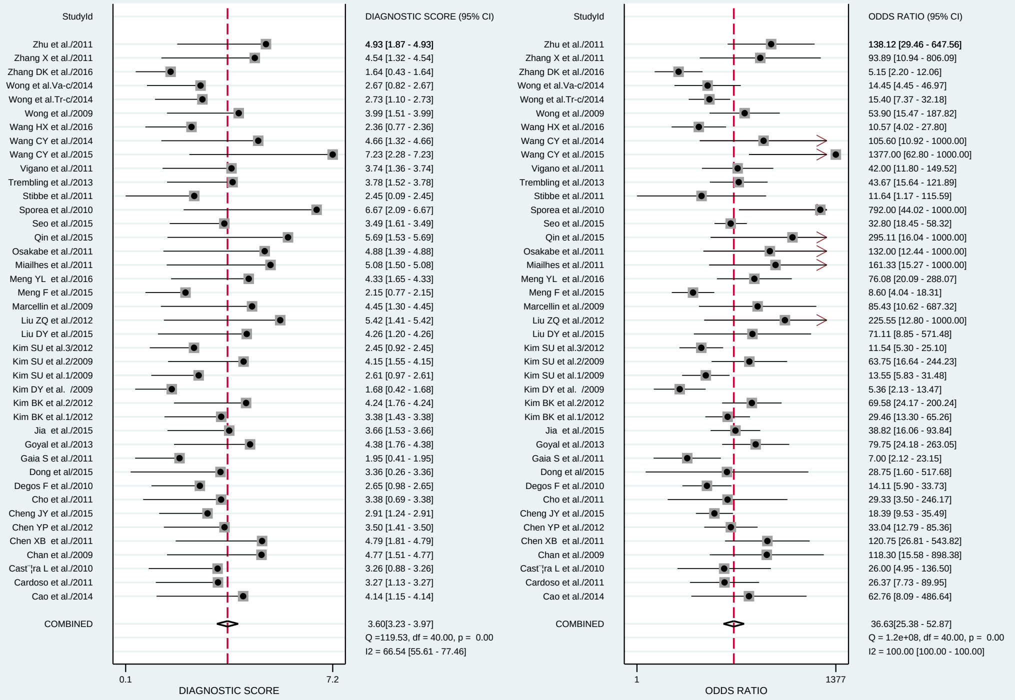
StudyId
DIAGNOSTIC SCORE (95% CI)
Zhu et al./2011 4.93 [1.87 - 4.93]
Zhang X et al./2011 4.54 [1.32 - 4.54]
Zhang DK et al./2016 1.64 [0.43 - 1.64]
Wong et al.Va-c/2014 2.67 [0.82 - 2.67]
Wong et al.Tr-c/2014 2.73 [1.10 - 2.73]
Wong et al./2009 3.99 [1.51 - 3.99]
Wang HX et al./2016 2.36 [0.77 - 2.36]
Wang CY et al./2014 4.66 [1.32 - 4.66]
Wang CY et al./2015 7.23 [2.28 - 7.23]
Vigano et al./2011 3.74 [1.36 - 3.74]
Trembling et al./2013 3.78 [1.52 - 3.78]
Stibbe et al./2011 2.45 [0.09 - 2.45]
Sporea et al./2010 6.67 [2.09 - 6.67]
Seo et al./2015 3.49 [1.61 - 3.49]
Qin et al./2015 5.69 [1.53 - 5.69]
Osakabe et al./2011 4.88 [1.39 - 4.88]
Miailhes et al./2011 5.08 [1.50 - 5.08]
Meng YL et al./2016 4.33 [1.65 - 4.33]
Meng F et al./2015 2.15 [0.77 - 2.15]
Marcellin et al./2009 4.45 [1.30 - 4.45]
Liu ZQ et al./2012 5.42 [1.41 - 5.42]
Liu DY et al./2015 4.26 [1.20 - 4.26]
Kim SU et al.3/2012 2.45 [0.92 - 2.45]
Kim SU et al.2/2009 4.15 [1.55 - 4.15]
Kim SU et al.1/2009 2.61 [0.97 - 2.61]
Kim DY et al. /2009 1.68 [0.42 - 1.68]
Kim BK et al.2/2012 4.24 [1.76 - 4.24]
Kim BK et al.1/2012 3.38 [1.43 - 3.38]
Jia et al./2015 3.66 [1.53 - 3.66]
Goyal et al./2013 4.38 [1.76 - 4.38]
Gaia S et al./2011 1.95 [0.41 - 1.95]
Dong et al/2015 3.36 [0.26 - 3.36]
Degos F et al./2010 2.65 [0.98 - 2.65]
Cho et al./2011 3.38 [0.69 - 3.38]
Cheng JY et al./2015 2.91 [1.24 - 2.91]
Chen YP et al./2012 3.50 [1.41 - 3.50]
Chen XB et al./2011 4.79 [1.81 - 4.79]
Chan et al./2009 4.77 [1.51 - 4.77]
Cast¨¦ra L et al./2010 3.26 [0.88 - 3.26]
Cardoso et al./2011 3.27 [1.13 - 3.27]
Cao et al./2014 4.14 [1.15 - 4.14]
COMBINED 3.60[3.23 - 3.97]
Q =119.53, df = 40.00, p = 0.00
I2 = 66.54 [55.61 - 77.46]
0.1
7.2
DIAGNOSTIC SCORE
StudyId
ODDS RATIO (95% CI)
Zhu et al./2011 138.12 [29.46 - 647.56]
Zhang X et al./2011 93.89 [10.94 - 806.09]
Zhang DK et al./2016 5.15 [2.20 - 12.06]
Wong et al.Va-c/2014 14.45 [4.45 - 46.97]
Wong et al.Tr-c/2014 15.40 [7.37 - 32.18]
Wong et al./2009 53.90 [15.47 - 187.82]
Wang HX et al./2016 10.57 [4.02 - 27.80]
Wang CY et al./2014 105.60 [10.92 - 1000.00]
Wang CY et al./2015 1377.00 [62.80 - 1000.00]
Vigano et al./2011 42.00 [11.80 - 149.52]
Trembling et al./2013 43.67 [15.64 - 121.89]
Stibbe et al./2011 11.64 [1.17 - 115.59]
Sporea et al./2010 792.00 [44.02 - 1000.00]
Seo et al./2015 32.80 [18.45 - 58.32]
Qin et al./2015 295.11 [16.04 - 1000.00]
Osakabe et al./2011 132.00 [12.44 - 1000.00]
Miailhes et al./2011 161.33 [15.27 - 1000.00]
Meng YL et al./2016 76.08 [20.09 - 288.07]
Meng F et al./2015 8.60 [4.04 - 18.31]
Marcellin et al./2009 85.43 [10.62 - 687.32]
Liu ZQ et al./2012 225.55 [12.80 - 1000.00]
Liu DY et al./2015 71.11 [8.85 - 571.48]
Kim SU et al.3/2012 11.54 [5.30 - 25.10]
Kim SU et al.2/2009 63.75 [16.64 - 244.23]
Kim SU et al.1/2009 13.55 [5.83 - 31.48]
Kim DY et al. /2009 5.36 [2.13 - 13.47]
Kim BK et al.2/2012 69.58 [24.17 - 200.24]
Kim BK et al.1/2012 29.46 [13.30 - 65.26]
Jia et al./2015 38.82 [16.06 - 93.84]
Goyal et al./2013 79.75 [24.18 - 263.05]
Gaia S et al./2011 7.00 [2.12 - 23.15]
Dong et al/2015 28.75 [1.60 - 517.68]
Degos F et al./2010 14.11 [5.90 - 33.73]
Cho et al./2011 29.33 [3.50 - 246.17]
Cheng JY et al./2015 18.39 [9.53 - 35.49]
Chen YP et al./2012 33.04 [12.79 - 85.36]
Chen XB et al./2011 120.75 [26.81 - 543.82]
Chan et al./2009 118.30 [15.58 - 898.38]
Cast¨¦ra L et al./2010 26.00 [4.95 - 136.50]
Cardoso et al./2011 26.37 [7.73 - 89.95]
Cao et al./2014 62.76 [8.09 - 486.64]
COMBINED 36.63[25.38 - 52.87]
Q = 1.2e+08, df = 40.00, p = 0.00
I2 = 100.00 [100.00 - 100.00]
1
1377
ODDS RATIO
