# Supplementary figures and images for: Transient Elastography for Significant Liver Fibrosis and Cirrhosis in Chronic Hepatitis B: A Meta-Analysis
Source: Can J Gastroenterol Hepatol. 2018 May 24;2018:3406789. doi: 10.1155/2018/3406789 (PMC5994263; doi:10.1155/2018/3406789)

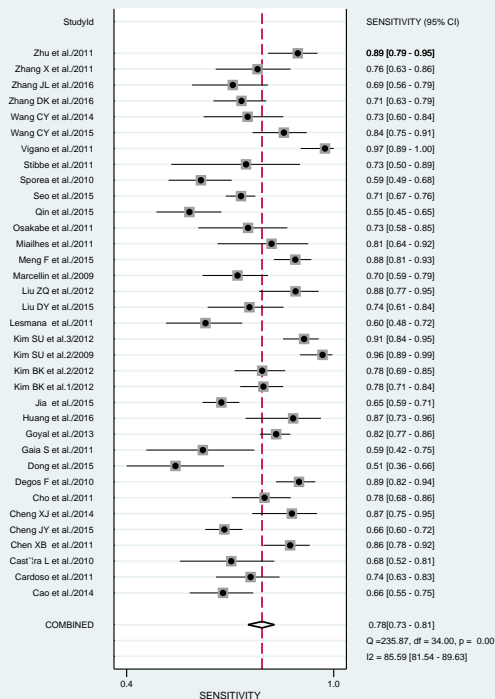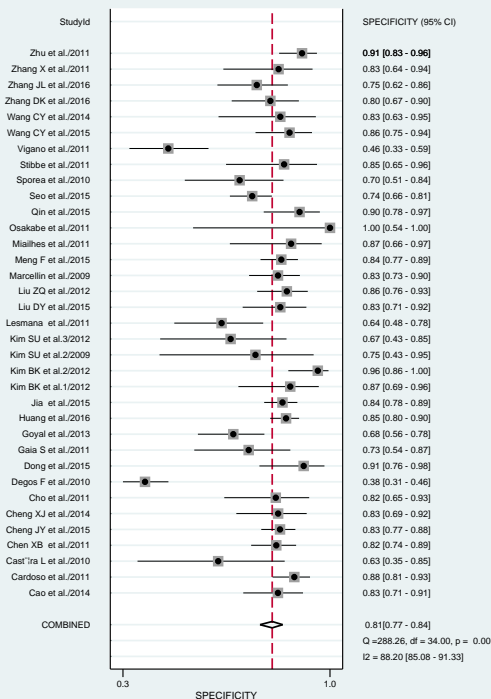

Supplement: Supplementary Materials — Supplementary Figure 1: meta-analysis of 32 studies that assessed the diagnosis accuracy of significant fibrosis (METAVIR F2–F4) based on transient elastography. A Forest plot of (A) sensitivity and specificity, (B) positive and negative likelihood ratio, and (C) diagnostic score (DS) and diagnostic odds ratio (DOR) for significant liver fibrosis (METAVIR F2–F4). Supplementary Figure 2: meta-analysis of 37 studies that assessed the diagnosis accuracy of cirrhosis (METAVIR F4) based on transient elastography. A Forest plot of (A) sensitivity and specificity, (B) positive and negative likelihood ratio, and (C) DS and DOR for cirrhosis (METAVIR F4). Supplementary Figure 3: Deeks' Funnel Plot Asymmetry Test for (A) significant fibrosis (METAVIR F2–F4) and (B) cirrhosis (METAVIR F4). [file 3406789.f1.zip › 3406789.f1.pdf]

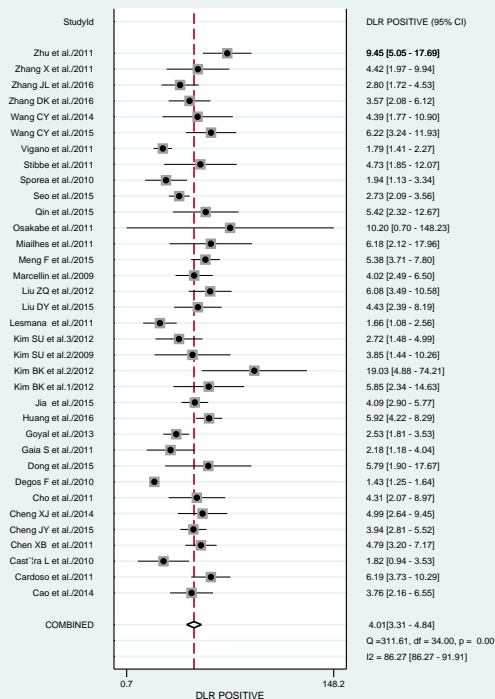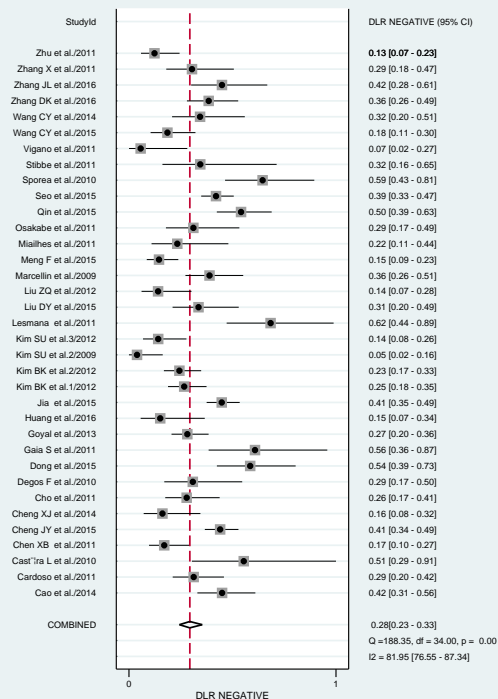

Supplement: Supplementary Materials — Supplementary Figure 1: meta-analysis of 32 studies that assessed the diagnosis accuracy of significant fibrosis (METAVIR F2–F4) based on transient elastography. A Forest plot of (A) sensitivity and specificity, (B) positive and negative likelihood ratio, and (C) diagnostic score (DS) and diagnostic odds ratio (DOR) for significant liver fibrosis (METAVIR F2–F4). Supplementary Figure 2: meta-analysis of 37 studies that assessed the diagnosis accuracy of cirrhosis (METAVIR F4) based on transient elastography. A Forest plot of (A) sensitivity and specificity, (B) positive and negative likelihood ratio, and (C) DS and DOR for cirrhosis (METAVIR F4). Supplementary Figure 3: Deeks' Funnel Plot Asymmetry Test for (A) significant fibrosis (METAVIR F2–F4) and (B) cirrhosis (METAVIR F4). [file 3406789.f1.zip › 3406789.f2.pdf]

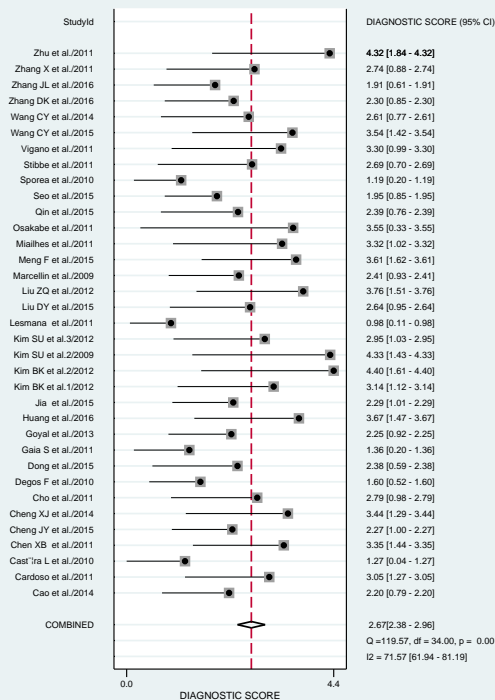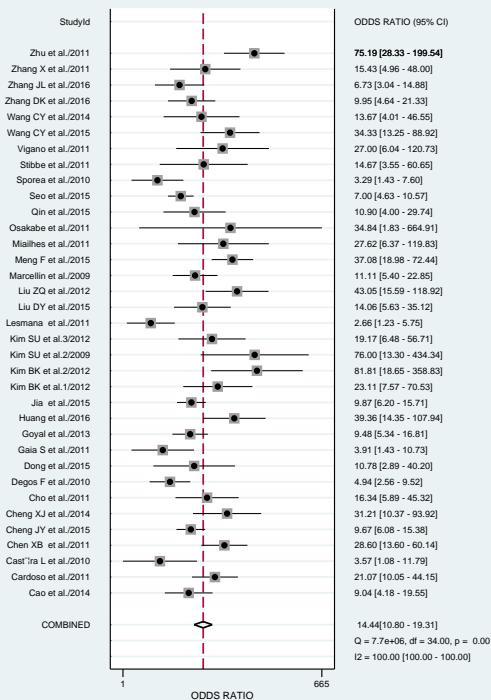

Supplement: Supplementary Materials — Supplementary Figure 1: meta-analysis of 32 studies that assessed the diagnosis accuracy of significant fibrosis (METAVIR F2–F4) based on transient elastography. A Forest plot of (A) sensitivity and specificity, (B) positive and negative likelihood ratio, and (C) diagnostic score (DS) and diagnostic odds ratio (DOR) for significant liver fibrosis (METAVIR F2–F4). Supplementary Figure 2: meta-analysis of 37 studies that assessed the diagnosis accuracy of cirrhosis (METAVIR F4) based on transient elastography. A Forest plot of (A) sensitivity and specificity, (B) positive and negative likelihood ratio, and (C) DS and DOR for cirrhosis (METAVIR F4). Supplementary Figure 3: Deeks' Funnel Plot Asymmetry Test for (A) significant fibrosis (METAVIR F2–F4) and (B) cirrhosis (METAVIR F4). [file 3406789.f1.zip › 3406789.f3.pdf]

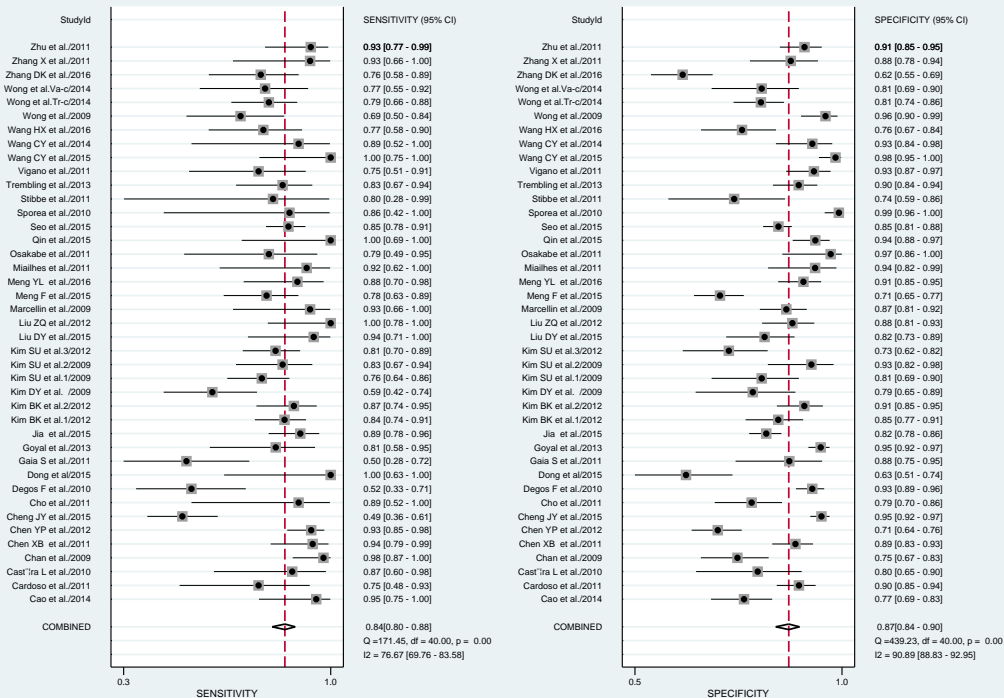

Supplement: Supplementary Materials — Supplementary Figure 1: meta-analysis of 32 studies that assessed the diagnosis accuracy of significant fibrosis (METAVIR F2–F4) based on transient elastography. A Forest plot of (A) sensitivity and specificity, (B) positive and negative likelihood ratio, and (C) diagnostic score (DS) and diagnostic odds ratio (DOR) for significant liver fibrosis (METAVIR F2–F4). Supplementary Figure 2: meta-analysis of 37 studies that assessed the diagnosis accuracy of cirrhosis (METAVIR F4) based on transient elastography. A Forest plot of (A) sensitivity and specificity, (B) positive and negative likelihood ratio, and (C) DS and DOR for cirrhosis (METAVIR F4). Supplementary Figure 3: Deeks' Funnel Plot Asymmetry Test for (A) significant fibrosis (METAVIR F2–F4) and (B) cirrhosis (METAVIR F4). [file 3406789.f1.zip › 3406789.f4.pdf]

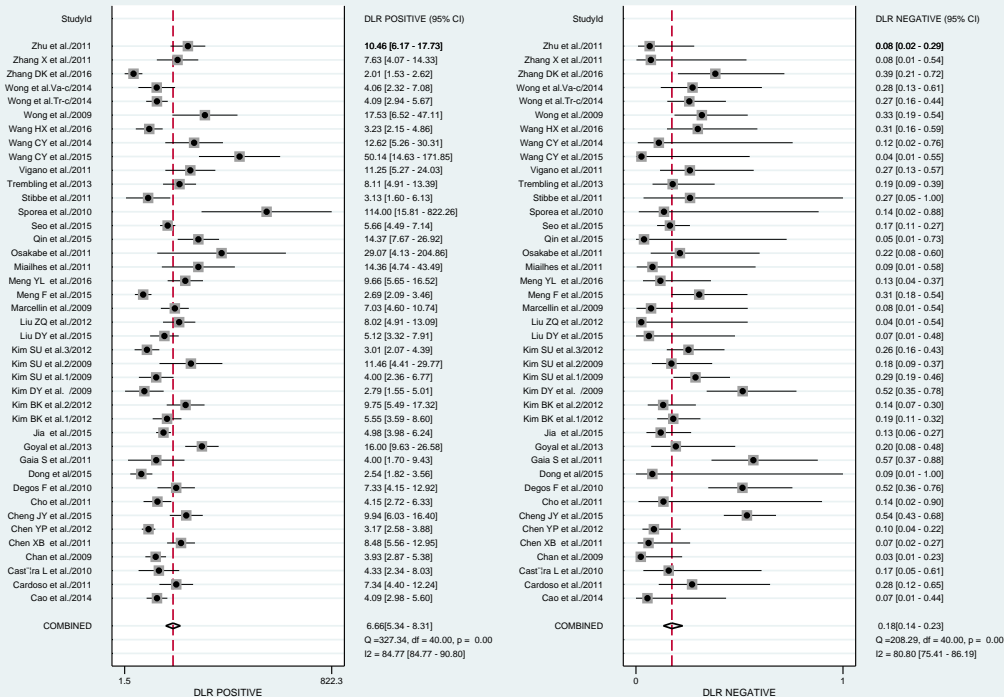

Supplement: Supplementary Materials — Supplementary Figure 1: meta-analysis of 32 studies that assessed the diagnosis accuracy of significant fibrosis (METAVIR F2–F4) based on transient elastography. A Forest plot of (A) sensitivity and specificity, (B) positive and negative likelihood ratio, and (C) diagnostic score (DS) and diagnostic odds ratio (DOR) for significant liver fibrosis (METAVIR F2–F4). Supplementary Figure 2: meta-analysis of 37 studies that assessed the diagnosis accuracy of cirrhosis (METAVIR F4) based on transient elastography. A Forest plot of (A) sensitivity and specificity, (B) positive and negative likelihood ratio, and (C) DS and DOR for cirrhosis (METAVIR F4). Supplementary Figure 3: Deeks' Funnel Plot Asymmetry Test for (A) significant fibrosis (METAVIR F2–F4) and (B) cirrhosis (METAVIR F4). [file 3406789.f1.zip › 3406789.f5.pdf]

Deeks' Funnel Plot Asymmetry Test  
pvalue = 0.26

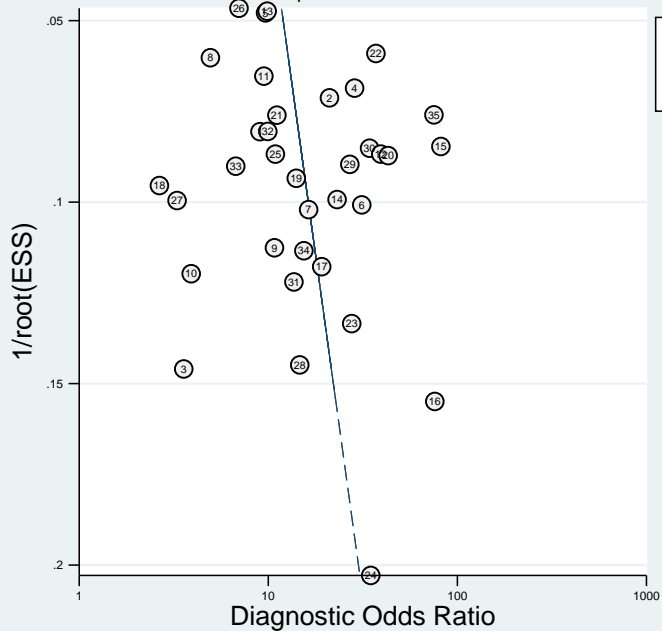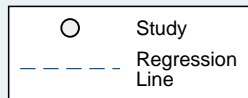

Supplement: Supplementary Materials — Supplementary Figure 1: meta-analysis of 32 studies that assessed the diagnosis accuracy of significant fibrosis (METAVIR F2–F4) based on transient elastography. A Forest plot of (A) sensitivity and specificity, (B) positive and negative likelihood ratio, and (C) diagnostic score (DS) and diagnostic odds ratio (DOR) for significant liver fibrosis (METAVIR F2–F4). Supplementary Figure 2: meta-analysis of 37 studies that assessed the diagnosis accuracy of cirrhosis (METAVIR F4) based on transient elastography. A Forest plot of (A) sensitivity and specificity, (B) positive and negative likelihood ratio, and (C) DS and DOR for cirrhosis (METAVIR F4). Supplementary Figure 3: Deeks' Funnel Plot Asymmetry Test for (A) significant fibrosis (METAVIR F2–F4) and (B) cirrhosis (METAVIR F4). [file 3406789.f1.zip › 3406789.f7.pdf]

Deeks' Funnel Plot Asymmetry Test  
pvalue = 0.02

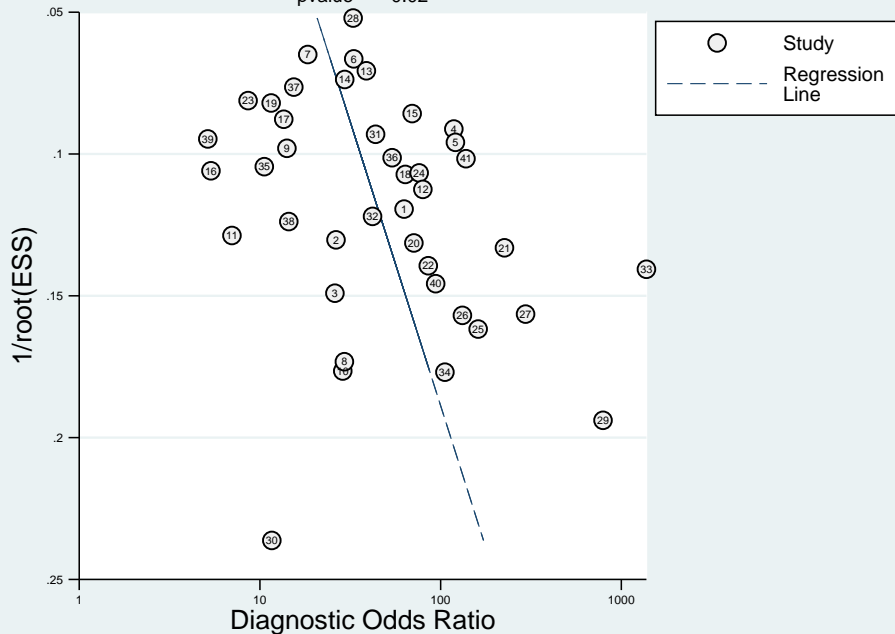

Supplement: Supplementary Materials — Supplementary Figure 1: meta-analysis of 32 studies that assessed the diagnosis accuracy of significant fibrosis (METAVIR F2–F4) based on transient elastography. A Forest plot of (A) sensitivity and specificity, (B) positive and negative likelihood ratio, and (C) diagnostic score (DS) and diagnostic odds ratio (DOR) for significant liver fibrosis (METAVIR F2–F4). Supplementary Figure 2: meta-analysis of 37 studies that assessed the diagnosis accuracy of cirrhosis (METAVIR F4) based on transient elastography. A Forest plot of (A) sensitivity and specificity, (B) positive and negative likelihood ratio, and (C) DS and DOR for cirrhosis (METAVIR F4). Supplementary Figure 3: Deeks' Funnel Plot Asymmetry Test for (A) significant fibrosis (METAVIR F2–F4) and (B) cirrhosis (METAVIR F4). [file 3406789.f1.zip › 3406789.f8.pdf]
